# In-depth summary of adverse events associated with Flurbiprofen: A real-world pharmacovigilance study from 2004 to 2024 using the FAERS database

**DOI:** 10.1371/journal.pone.0329636

**Published:** 2025-08-06

**Authors:** Chengkai Yang, Qian Guo, Yang Cheng, Fengjing Liu, Hui Zhang, Huaxiang Wang

**Affiliations:** 1 Clinical Oncology School of Fujian Medical University, Fujian Cancer Hospital, Fuzhou, China; 2 Department of Rhinology, The First Affiliated Hospital of Zhengzhou University, Zhengzhou, China; 3 Department of Hepatobiliary and Pancreatic Surgery, Taihe Hospital, Affiliated Hospital of Hubei University of Medicine, Shiyan, Hubei province, China; Deccan School of Pharmacy, INDIA

## Abstract

**Background:**

Flurbiprofen, as a widely used nonsteroidal anti-inflammatory drug (NSAID), is commonly employed to relieve mild to moderate pain and inflammation. Understanding its adverse reactions in real-world usage is of significant importance.

**Methods:**

Reports of all adverse drug events (ADEs) related to flurbiprofen were extracted from the FAERS database, covering the period from Q1 2004 to Q3 2024. These reports were standardized and analyzed using various signal quantification techniques, including Reporting Odds Ratios (ROR), Proportional Reporting Ratios (PRR), Bayesian Confidence Propagation Neural Network (BCPNN), and Multi-item Gamma Poisson Shrinkage (MGPS). Finally, the association between flurbiprofen and ADEs as well as clinical medical events was assessed.

**Results:**

A total of 275 cases from the target population were identified in the FAERS database, with 788 instances of adverse events (AEs) occurring across 46 organ systems. We identified not only some common adverse reactions listed in the drug’s package insert, such as acute kidney injury, nausea and vomiting, and facial edema, but also significant signals that were not mentioned in the package insert, including Dysphonia, Drug abuse, and Pancreatitis acute. The median time to onset of flurbiprofen-related AEs was 1 day (interquartile range [IQR] 0–5 days), with most AEs occurring within the first month of flurbiprofen use.

**Conclusion:**

This study confirmed some common adverse reactions listed in the flurbiprofen drug package insert and identified significant unexpected adverse reactions. These findings can assist clinicians in conducting more comprehensive clinical monitoring when using the drug, thereby ensuring patient safety during treatment.

## 1. Introduction

Flurbiprofen is a widely used nonsteroidal anti-inflammatory drug (NSAID) known for its efficacy in treating various conditions, including osteoarthritis, rheumatoid arthritis and a variety of pain, fever and other symptoms [1]. It is commonly prescribed for conditions such as osteoarthritis, rheumatoid arthritis, and other musculoskeletal disorders, as well as for short-term pain relief from dental or post-surgical procedures [2]. Like all NSAIDs, flurbiprofen exerts its therapeutic effects primarily by inhibiting cyclooxygenase enzymes (COX-1 and COX-2), which are involved in the synthesis of prostaglandins that mediate inflammation, pain, and fever [3]. However, despite its therapeutic benefits, flurbiprofen, like other NSAIDs, is associated with a range of potential adverse drug events (ADEs), which can impact patient safety. A systematic review of 26 studies showed that adverse events associated with flurbiprofen 8.75 mg treatment mainly included nervous system (e.g., nausea, abdominal pain), digestive system (e.g., headache, dizziness) and allergic reactions [4].

Understanding the adverse event profile of flurbiprofen in real-world settings is crucial for improving clinical decision-making and patient care. While the drug’s package insert lists several known side effects, real-world pharmacovigilance data can provide a more comprehensive understanding of its safety profile, uncovering less common or unexpected adverse events that may not be documented in clinical trials.

Pharmacovigilance plays a vital role in monitoring the safety of pharmaceuticals after they are marketed, and the Food and Drug Administration Adverse Event Reporting System (FAERS) database is one of the key resources for such analysis. The FAERS database collects and stores reports of ADEs submitted by healthcare providers, patients, and pharmaceutical companies, offering valuable insights into the safety of drugs over time and across diverse patient populations [5–7]. However, adverse events associated with flurbiprofen have not been thoroughly explored through this vast real-world dataset.

This study aims to provide an in-depth summary of the adverse events associated with flurbiprofen from Q1 2004 to Q3 2024, using the FAERS database. By analyzing the frequency, severity, and type of ADEs, as well as identifying new or previously underreported adverse reactions, this study aims to enhance the understanding of the safety profile of flurbiprofen, providing clinicians with safer and more comprehensive medication guidance.

## 2. Materials and methods

### 2.1. Data sources, management, and study design

**Fig 1:**
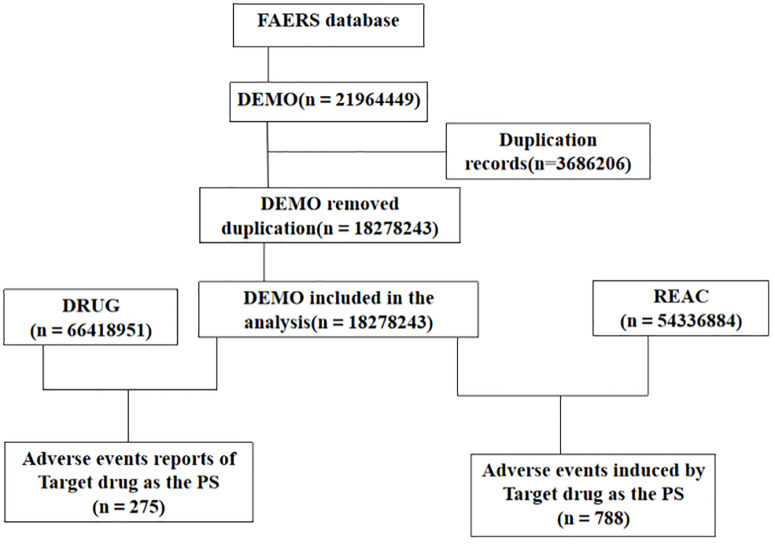
Detailed flowchart of this study.

This study is a retrospective pharmacovigilance analysis using the U.S. Food and Drug Administration Adverse Event Reporting System (FAERS) database. The FAERS database contains several quarterly data files, including: DEMO (Demographic): Contains patient demographic information (e.g., age, gender, weight) and administrative case details. DRUG (Drug/Biologic): Lists all medications reported for each case, including suspect/concomitant drugs and their administration routes. REAC (Reaction): Documents all adverse event terms (MedDRA preferred terms) reported for each case. All adverse event reports that identified flurbiprofen as the primary suspect drug were compiled. The analysis covered the period from Q1 2024 to Q3 2024. Due to the ongoing updates to the FAERS database, duplicate reports of adverse drug events (ADEs) may exist. Therefore, we followed FDA-recommended practices to handle duplicates. For reports with the same case identifier (CASEID), the report with the most recent FDA receipt date (FDA_DT) was retained. If CASEID was the same, the report with the latest FDA_DT was chosen; if both CASEID and FDA_DT were identical, the report with the higher PRIMARYID was selected [8]. The adverse event terms were standardized using the MedDRA (Medical Dictionary for Regulatory Activities) version 27.1.In total, we obtained 18,278,243 DEMO reports, 66,418,951 DRUG cases, and 54,336,884 REAC records. Fig 1 presents the detailed flowchart of this study.

### 2.2. Statistical analysis

In pharmacovigilance studies, disproportionality analysis is an effective tool for identifying adverse drug reaction signals. To enhance the reliability of this study, four disproportionality analysis methods were used to detect the association between flurbiprofen and adverse events, including the reporting odds ratio (ROR), proportional reporting ratio (PRR), Bayesian confidence propagation neural network (BCPNN), and Multi-item Gamma Poisson Shrinker (MGPS). Values above the threshold indicate stronger signal strength, with larger values representing stronger signals. The onset time of adverse events related to flurbiprofen was defined as the interval between the initiation of flurbiprofen treatment and the occurrence of the adverse event. The Weibull distribution was used to model the incidence of adverse events over time. All statistical analyses in this study were performed using SAS 9.4.

### 2.3. Ethics statement

This study was conducted using publicly available, de-identified data from the FDA Adverse Event Reporting System (FAERS) database. As no identifiable personal information was accessed and no direct patient involvement occurred, ethical approval was not required.

## 3. Results

### 3.1. Descriptive characteristics

**Fig 2:**
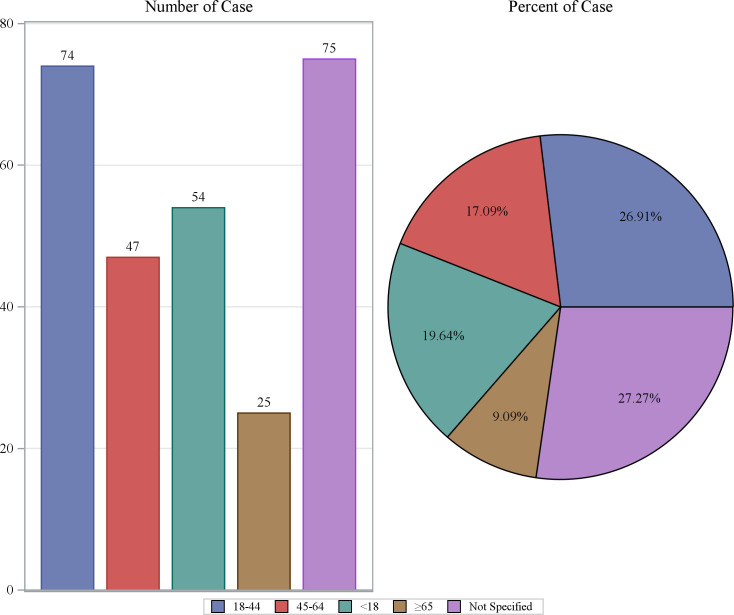
Age distribution of AE reports.

**Fig 3:**
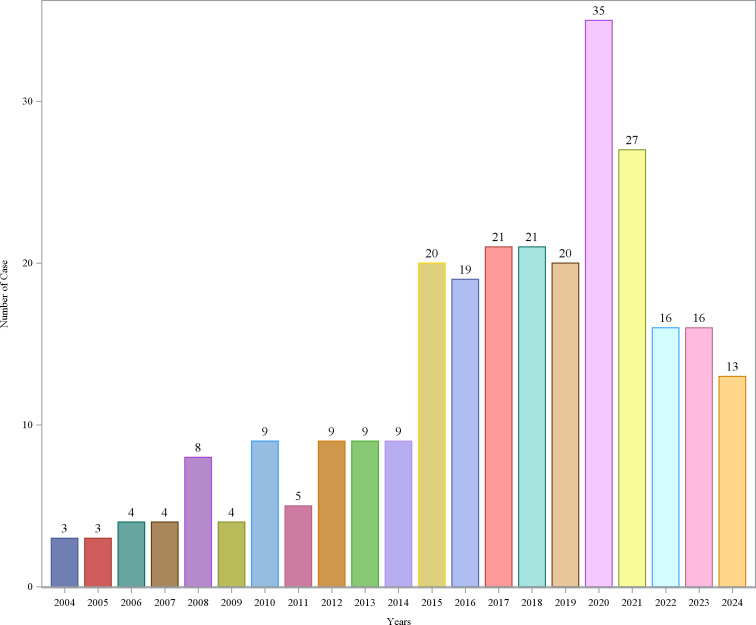
Annual distribution of AE reports.

**Fig 4:**
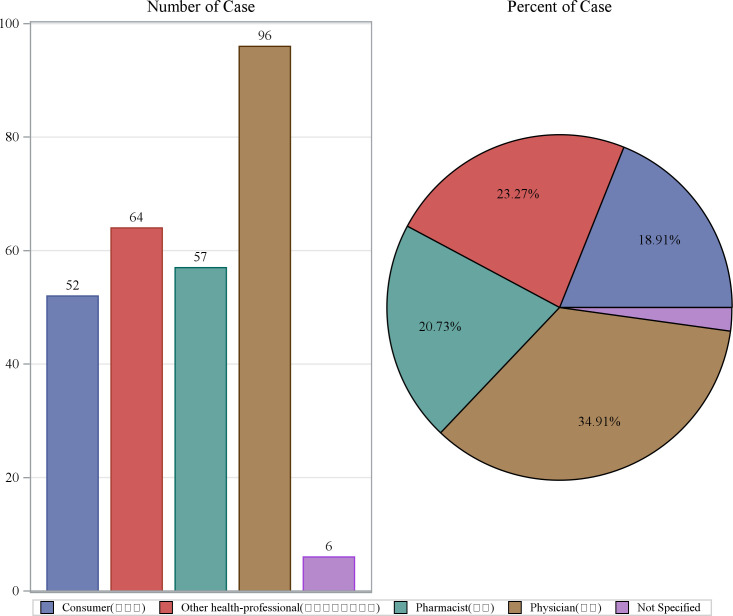
Reporter distribution of AE reports.

**Fig 5:**
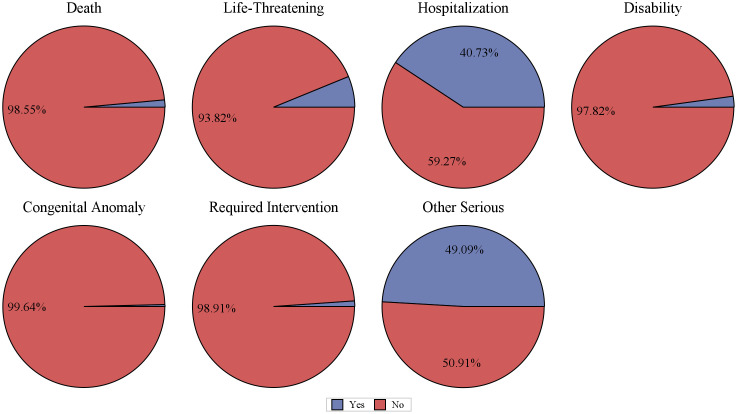
Outcomes report distribution of AE reports.

From Q1 2004 to Q3 2024, a total of 83 quarters were included in the analysis, with a background patient population of 18,278,243 (with 54,336,884 adverse event reports). Among them, the flurbiprofen patient population comprised 275 individuals (with 788 adverse event reports). The demographic characteristics of the flurbiprofen-related ADEs are shown in Table 1. In terms of gender, the incidence rate of adverse events in females was 68.73%, while in males it was 22.18%. The highest proportion of AE reports came from France (36.00%), followed by the United States (19.27%), Italy (18.91%), Turkey (10.55%), and the United Kingdom (5.45%). The majority of adverse events occurred within 0–30 days after medication (40.73%), with the median time of occurrence being 1 day after drug administration. The median weight of the included patients was 63.5 kg. Regarding age, the highest number of reports came from patients aged 18–44 years (26.91%), followed by those under 18 years (19.64%), with the lowest number of reports from patients over 65 years (9.09%) (Fig 2). The year with the highest number of AE reports was 2020 (12.73%), followed by 2021 (9.82%), and 2017 and 2018 (both 7.64%) (Fig 3). Most reports were submitted by healthcare professionals, with physicians, other healthcare professionals, and pharmacists accounting for 34.91%, 23.27%, and 20.73%, respectively, which significantly enhances the reliability of this study (Fig 4). Severe reports accounted for 86.18%, with nearly half of the outcomes classified as other serious outcomes (49.09%), followed by hospitalization (40.73%), life-threatening events (6.18%), and death (1.45%) (Fig 5).

**Table 1:** Demographic characteristics associated with flurbiprofen-related adverse drug reactions from the FAERS database (January 2004 to September 2024).

Indicators	Case (%)
Sex	
Female(%)	189(68.73)
Male(%)	61(22.18)
Not Specified(%)	25(9.09)
Reported countries, n (%)	
France(%)	99(36.00)
United States of America(%)	53(19.27)
Italy(%)	52(18.91)
Turkey(%)	29(10.55)
United Kiongdom(%)	15(5.45)
Outcomes	
Serious(%)	237(86.18)
Non-Serious(%)	38(13.82)
Time-to-onset (days)	
0-30d(%)	112(40.73)
31-360d(%)	4(1.81)
>360d(%)	5(1.82)
N(Missing)(%)	153(55.64)
Time-to-onset (days)	
N(Missing)	122(153)
Mean(SD)	100.43(659.89)
Median(Q1,Q3)	1.00(0.00,5.00)
Min,Max	0.00,6879.00
Weight (Kg)	
N(Missing)	79(196)
Mean(SD)	70.93(24.38)
Median(Q1,Q3)	63.50(56.00,80.00)
Min,Max	41.30,196.00

### 3.2. Distribution and intensity of flurbiprofen AEs at SOC level

**Fig 6:**
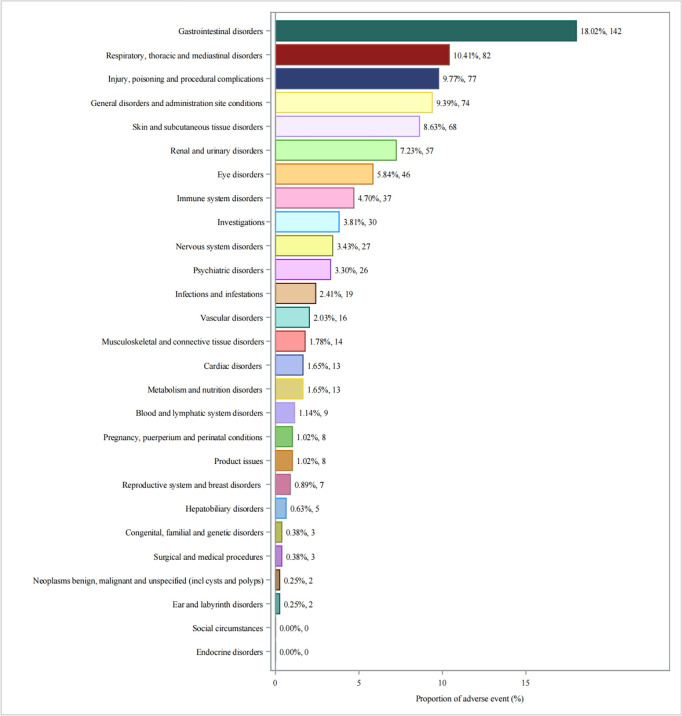
Reported cases of ADEs per SOC level.

Flurbiprofen related AEs were mainly distributed among 25 different system organ classes (SOCs), the number of case reports for the associated SOC is shown in Fig 6. Of these, the top five SOCs were Gastrointestinal disorders (n = 142, 18.02%), Respiratory, thoracic and mediastinal disorders (n = 82, 10.41%), Injury, poisoning and procedural complications (n = 77, 9.77%), General administration and subcutaneous site conditions (n = 74, 9.39%), and Skin and tissue disorders (n = 68, 8.63%). with the comprehensive details of the remaining SOCs available in Table 2.

**Table 2:** Signal intensity of AEs at SOC level for flurbiprofen in FAERS database.

SOC	Case number(n)	Case proportion(%)
Gastrointestinal disorders*	142	18.02
Respiratory, thoracic and mediastinal disorders*	82	10.41
Injury, poisoning and procedural complications*	77	9.77
General disorders and administration site conditions*	74	9.39
Skin and subcutaneous tissue disorders*	68	8.63
Renal and urinary disorders*	57	7.23
Eye disorders*	46	5.84
Immune system disorders*	37	4.70
Investigations*	30	3.81
Nervous system disorders	27	3.43
Psychiatric disorders	26	3.30
Infections and infestations	19	2.41
Vascular disorders*	16	2.03
Musculoskeletal and connective tissue disorders	14	1.78
Metabolism and nutrition disorders	13	1.65
Cardiac disorders	13	1.65
Blood and lymphatic system disorders	9	1.14
Product issues	8	1.02
Pregnancy, puerperium and perinatal conditions	8	1.02
Reproductive system and breast disorders	7	0.89
Hepatobiliary disorders	5	0.63
Surgical and medical procedures	3	0.38
Congenital, familial and genetic disorders	3	0.38
Ear and labyrinth disorders	2	0.25
Neoplasms benign, malignant and unspecified (incl cysts and polyps)	2	0.25

* SOCs that met all four criteria simultaneously. SOC, system organ class; PT, preferred term; ROR, reporting odds ratio; CI, confidence interval; PRR, proportional reporting ratio; χ^2^, chi-information component; IC, information component; IC025, the lower limit of 95% CI of the IC; EBGM, empirical Bayesian geometric mean; EBGM05, the lower limit of 95% CI of EBGM.

### 3.3. Analysis of Adverse Reaction Frequency

**Fig 7:**
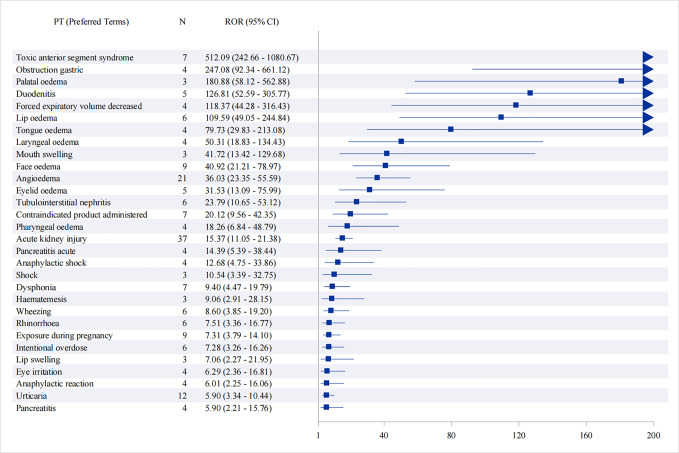
Top 50 Signal strength of adverse events at the PTs ranked by ROR.

Suspected signals for flurbiprofen were detected by four pharmacovigilance algorithms (ROR, PRR, BCPNN and MGPS) with a total of 37 PTs that met four algorithms involving 11 SOCs, and then in descending order of frequency, we identified adverse reactions commonly observed in the labeling of flurbiprofen, such as acute kidney injury, angioedema, abdominal discomfort, anaphylaxis, and Urticaria. In addition, we unexpectedly identified off-label adverse reactions, including Dysphonia, Drug abuse, and Pancreatitis acute, which can be found in Table 3. Fig 7 shows the ROR and its 95% interval for signal intensities of the first 50 flurbiprofen-associated PTs.

**Table 3:** Signal intensity reported for flurbiprofen AEs at the preferred term (PT) level.

Preferred Terms	Case	ROR(95% CI)	PRR(Chi-Square)	IC(IC-2SD)	EBGM(EBGM05)
Acute kidney injury	37	15.37(11.05–21.38)	14.69(473.51)	3.88(2.95)	14.69(10.56)
Angioedema	21	36.03(23.35–55.59)	35.09(695.76)	5.13(3.16)	35.08(22.74)
Abdominal pain upper	14	5.48(3.23–9.30)	5.40(50.41)	2.43(1.31)	5.40(3.19)
Hypersensitivity	13	5.61(3.24–9.70)	5.53(48.37)	2.47(1.28)	5.53(3.20)
Urticaria	12	5.90(3.34–10.44)	5.83(48.12)	2.54(1.28)	5.83(3.30)
Drug hypersensitivity	11	4.40(2.43–7.98)	4.35(28.50)	2.12(0.93)	4.35(2.40)
Face oedema	9	40.92(21.21–78.97)	40.47(346.34)	5.34(2.11)	40.45(20.96)
Exposure during pregnancy	9	7.31(3.79–14.10)	7.24(48.46)	2.86(1.24)	7.24(3.75)
Dysphagia	7	5.56(2.64–11.71)	5.52(25.96)	2.47(0.79)	5.52(2.62)
Dysphonia	7	9.40(4.47-19.79)	9.33(52.10)	3.22(1.17)	9.33(4.43)
Toxic anterior segment syndrome	7	512.09(242.66–1080.67)	507.55(3513.07)	8.98(1.95)	503.85(238.76)
Contraindicated product administered	7	20.12(9.56–42.35)	19.95(126.02)	4.32(1.54)	19.94(9.48)
Drug abuse	6	5.60(2.51–12.50)	5.56(22.48)	2.48(0.66)	5.56(2.49)
Intentional overdose	6	7.28(3.26–16.26)	7.23(32.27)	2.85(0.84)	7.23(3.24)
Lip oedema	6	109.59(49.05–244.84)	108.76(639.66)	6.76(1.63)	108.59(48.61)
Rhinorrhoea	6	7.51(3.36–16.77)	7.46(33.60)	2.90(0.86)	7.46(3.34)
Wheezing	6	8.60(3.85–19.20)	8.54(39.98)	3.09(0.94)	8.54(3.82)
Tubulointerstitial nephritis	6	23.79(10.65–53.12)	23.62(129.95)	4.56(1.38)	23.61(10.57)
Duodenitis	5	126.81(52.59–305.77)	126.01(619.01)	6.97(1.34)	125.79(52.17)
Eyelid oedema	5	31.53(13.09–75.99)	31.34(146.82)	4.97(1.19)	31.33(13.00)
Anaphylactic reaction	4	6.01(2.25–16.06)	5.99(16.62)	2.58(0.29)	5.98(2.24)
Anaphylactic shock	4	12.68(4.75–33.86)	12.62(42.80)	3.66(0.63)	12.62(4.72)
Eye irritation	4	6.29(2.36–16.81)	6.26(17.71)	2.65(0.31)	6.26(2.35)
Forced expiratory volume decreased	4	118.37(44.28–316.43)	117.77(462.34)	6.88(0.98)	117.57(43.98)
Laryngeal oedema	4	50.31(18.83–134.43)	50.06(192.19)	5.64(0.92)	50.02(18.72)
Medication error	4	5.68(2.13–15.18)	5.66(15.36)	2.50(0.26)	5.66(2.12)
Nasal congestion	4	5.57(2.09–14.88)	5.55(14.93)	2.47(0.24)	5.55(2.08)
Obstruction gastric	4	247.08(92.34–661.12)	245.83(971.88)	7.94(1.00)	244.96(91.55)
Pancreatitis	4	5.90(2.21–15.76)	5.88(16.20)	2.55(0.28)	5.88(2.20)
Pancreatitis acute	4	14.39(5.39–38.44)	14.32(49.58)	3.84(0.67)	14.32(5.36)

SOC, system organ class; PT, preferred term; ROR, reporting odds ratio; CI, confidence interval; PRR, proportional reporting ratio; χ^2^, chi-information component; IC, information component; IC025, the lower limit of 95% CI of the IC; EBGM, empirical Bayesian geometric mean; EBGM05, the lower limit of 95% CI of EBGM. *: The instruction does not mention.

Note1: ranked by Reports.

Note2: Signals are detected when all the following criteria are met:a ≥ 3, PRR ≥2 and Chi-Square ≥ 4, lower limit of 95% CI of ROR > 1, IC025 > 0, EBGM05 > 2.

### 3.4. Time to onset of flurbiprofen related adverse events

**Fig 8:**
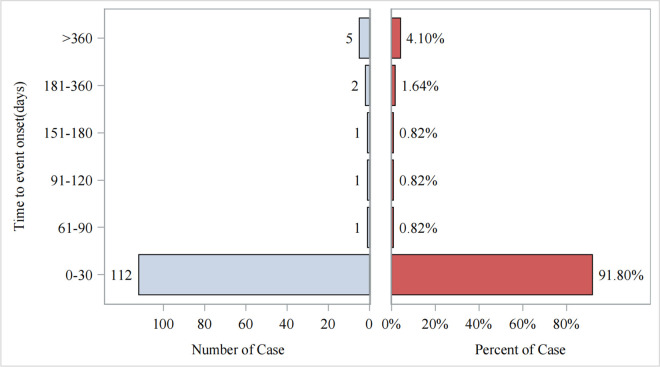
Time to onset of flurbiprofen-related AEs.

As shown in Fig 8, the time to onset of AEs was mainly concentrated in the first month after the start of flurbiprofen treatment (n = 112, 91.8%), with a median time to onset of 1 day (interquartile range [IQR] 0–5 days).

## Discussion

Previous studies on flurbiprofen have mainly focused on the drug’s mechanism of action and clinical efficacy [9–11], with very few large-scale real-world studies. This study is the first to conduct an in-depth analysis of flurbiprofen-related adverse drug events (ADEs) based on the FAERS database, revealing various safety risks associated with its use. These findings provide new safety warnings for clinical use and help better identify potential risks related to flurbiprofen, offering valuable guidance for doctors to make informed prescribing decisions.

Flurbiprofen is a class of nonsteroidal anti-inflammatory drugs (NSAIDs), and gastrointestinal organ damage is the most common adverse effect of NSAIDs. A prospective, cross-sectional, multicenter study in India showed that the incidence of gastrointestinal complications associated with NSAIDs was as high as 30.08% [12].Various degrees of gastric mucosal loss have also been observed in patients with class risk arthritis and pain treated with NSAIDs [13].A retrospective analysis of 820 patients showed that among patients who underwent upper gastrointestinal (GI) endoscopy, the proportion of patients with NSAID-associated peptic ulcer was 14.7%, with duodenal ulcer being more common than gastric ulcer [14].Our results also showed that the gastrointestinal system was the main organ involved in flurbiprofen-related adverse reactions, manifested as nausea and vomiting, abdominal pain and diarrhea, upper abdominal pain and other symptoms. This may be that NSAIDs inhibit the gastroprotective factors PGE2 and PGI2, which in turn cause damage to the gastric mucosa [15], which suggests that when such drugs are used in our clinical practice, we need to guard against gastrointestinal adverse reactions in patients and intervene in time to avoid more serious consequences.

Another common adverse effect of NSAIDs is renal injury [16], which was also confirmed in our study.The main mechanism of action of NSAIDs is inhibition of cyclooxygenase (COX) enzymes, which interferes with the conversion of arachidonic acid to prostacyclin, prostaglandin E2, and thromboxane, among which prostaglandins can dilate renal vessels and increase renal perfusion. Acute kidney injury (AKI) can be caused by glomerular hypoperfusion when NSAIDs are used [17].Results of a real-world analysis based on the FAERS database showed that NSAID-associated kidney injury occurred early in drug therapy, a phenomenon that is more prominent in elderly patients [18].Some studies have shown that NSAIDs use in children may have more severe consequences of acute renal failure, which may be associated with acute tubulointerstitial nephritis (ATIN) [19,20].NSAID associated nephropathy is reported to be diagnosed in approximately 2.5 million people each year in the United States [21].This finding further suggests that patients should be closely monitored for changes in renal function when using flurbiprofen, especially in specific predisposed patient groups (those with renal insufficiency, children, and the elderly).We also found that many patients experienced difficulty breathing after using flurbiprofen, a reaction that has been reported in other studies as well [22–24]. Therefore, it is important to closely monitor patients for signs of respiratory distress after administration. In such cases, bronchodilators can be used for symptomatic treatment, and discontinuation of the drug should be considered if necessary.

Our analysis identified angioedema as the second most frequent adverse event, a finding consistent with prior reports implicating NSAIDs in hypersensitivity reactions [25]. Angioedema is a potentially life-threatening condition characterized by rapid swelling of the dermis and submucosal tissues, often involving the face, lips, or airways. The mechanism may involve bradykinin accumulation (via cyclooxygenase-1 inhibition and prostaglandin E2 suppression) or mast cell-mediated histamine release, particularly in patients with predisposing factors such as a history of NSAID intolerance or chronic urticaria [26,27].

This study is the first to identify several notable adverse event signals related to flurbiprofen that are not listed in the drug’s prescribing information, including acute pancreatitis, intentional overdose, and dysphonia. These findings suggest that flurbiprofen may, in certain special cases, cause rare and severe clinical issues, particularly acute pancreatitis. Although rare, its potentially fatal nature requires clinicians to pay close attention to the patient’s medical history and clinical manifestations during flurbiprofen use. As flurbiprofen is commonly used as a painkiller, many patients may have a tendency to intentionally overdose, which also necessitates strict adherence to dosing guidelines to prevent the development of addictive behaviors.

Additionally, our findings show that the median onset time for flurbiprofen-related adverse events is 1 day (interquartile range [IQR] 0–5 days), with most adverse events occurring within the first month of flurbiprofen use. This finding further indicates that flurbiprofen may cause more acute safety issues in the short term. Therefore, clinicians should pay special attention to the early stages of drug use in order to identify and intervene in adverse events at an early stage.

Although this study is based on real-world data from the FAERS database and provides valuable drug safety information, it still has some limitations. This study has several limitations. Reporting bias and data omissions in the FAERS database may affect the completeness and accuracy of the adverse event records. Additionally, the database provides only broad reporter categories (e.g., physician, pharmacist, consumer) without further detail on clinical specialty or setting, limiting the granularity of our analysis. Moreover, FAERS lacks detailed patient-level clinical information, such as comorbidities, medical history, cancer status, or prior treatments, which prevents us from distinguishing between relatively healthy patients and those with complex medical conditions. These constraints underscore the need for future studies incorporating more comprehensive clinical data, such as prospective cohort studies or randomized controlled trials, to further validate the safety profile of flurbiprofen.

## Conclusion

This study is the first to conduct an in-depth analysis of flurbiprofen-related adverse events based on the FAERS database, revealing various safety risks associated with its use in the real world. We confirmed some common adverse reactions listed on the drug label, while also identifying significant signals not mentioned in the prescribing information, including acute pancreatitis, intentional overdose, and dysphonia. Most of these events occurred within the first month of drug use, highlighting the potential for acute safety issues in the short term.

## Supporting information

S1 File Date. (XLSX)
